# Decreased Tissue COX5B Expression and Mitochondrial Dysfunction during Sepsis-Induced Kidney Injury in Rats

**DOI:** 10.1155/2017/8498510

**Published:** 2017-01-26

**Authors:** Jochen Hinkelbein, Lennert Böhm, Stefan Braunecker, Christoph Adler, Edoardo De Robertis, Fabrizio Cirillo

**Affiliations:** ^1^Department of Anaesthesiology and Intensive Care Medicine, University Hospital Cologne, Kerpener Str. 62, 50937 Cologne, Germany; ^2^Department of Cardiology, University Hospital Cologne, Kerpener Str. 62, 50937 Cologne, Germany; ^3^Department of Neurosciences, Reproductive and Odontostomatological Sciences, University of Naples “Federico II”, Via S. Pansini, No. 5, 80131 Napoli, Italy

## Abstract

*Background*. Sepsis is defined as a life-threatening organ dysfunction due to a dysregulated host response to infection. Sepsis is the dominant cause of acute kidney injury (AKI), accounting for nearly 50% of episodes of acute renal failure. Signaling cascades and pathways within the kidney are largely unknown and analysis of these molecular mechanisms may enhance knowledge on pathophysiology and possible therapeutic options.* Material and Methods*. 26 male Wistar rats were assigned to either a sham group (control, *N* = 6) or sepsis group (*N* = 20; cecal ligature and puncture model, 24 and 48 hours after CLP). Surviving rats (*n* = 12) were decapitated at 24 hours (early phase; *n* = 6) or 48 hours (late phase; *n* = 6) after CLP and kidneys removed for proteomic analysis. 2D-DIGE and DeCyder 2D software (*t*-test, *P* < 0.01) were used for analysis of significantly regulated protein spots. MALDI-TOF in combination with peptide mass fingerprinting (PMF) as well as Western Blot analysis was used for protein identification. Bioinformatic network analyses (STRING, GeneMania, and PCViz) were used to describe protein-protein interactions.* Results*. 12 spots were identified with significantly altered proteins (*P* < 0.01) in the three analyzed groups. Two spots could not be identified. Four different proteins were found significantly changed among the groups: major urinary protein (MUP5), cytochrome c oxidase subunit B (COX5b), myosin-6 (MYH6), and myosin-7 (MYH7). A significant correlation with the proteins was found for mitochondrial energy production and electron transport.* Conclusions*. COX5B could be a promising biomarker candidate since a significant association was found during experimental sepsis in the present study. For future research, COX5B should be evaluated as a biomarker in both human urine and serum to identify sepsis.

## 1. Introduction

Sepsis has been recently defined as a life-threatening organ dysfunction due to a dysregulated host response to infection [1]. It is characterized by a specific set of systemic reactions to overwhelming infection [1, 2]. During clinical course, sepsis is often seriously complicated by renal dysfunction and triggers high mortality [3, 4], being the leading cause of death in critically ill patients [5].

The kidney shows extreme metabolic activity and an adequate energy supply is a prerequisite for its multiple functions [6]. Furthermore, sepsis is the dominant cause of acute kidney injury (AKI), accounting for nearly 50% of episodes of acute renal failure [7, 8]. AKI has been a common and severe problem in critically ill patients during the last decade [9]. Between 5% and 20% of critically ill patients in the intensive care unit (ICU) have at least an episode of AKI [9]. Early identification of those patients at risk for AKI could help clinical decision making [10].

Acute reduction in renal function during sepsis arises from a combination of local ischemia, inflammation, and additional factors [8, 11]. Sepsis-affected signaling cascades and pathways within the kidney are largely unknown. More than 1 million different protein variants are present in the mammalian organism, whose cellular abundance, functional status, and localization vary over time and are influenced by numerous environmental interactions [12]. However, analysis of these molecular mechanisms may enhance knowledge on pathophysiology and possible therapeutic options but may also suggest possible biomarker candidates to identify sepsis early in the clinical course. Unfortunately, patients' kidneys are typically not usable for those analyses. Consequently, animal models are commonly used as replace.

During the last decade, proteomics has increasingly been applied to identify and quantify mitochondrial proteins related to cellular perturbations, which enforce and elaborate data from metabolites and gene sequences, for example, in human mitochondrial disorders [12]. A proteomic approach for identification, quantification, and characterization of proteins of interest in biological materials facilitates understanding the pathogenesis. This approach has been extensively used for several diseases, for example, in sepsis [13–16], hyperoxia [17–19], cancer [20], liver diseases [20, 21], cardiac diseases [22, 23], neurodegenerative diseases [24, 25], and renal research [26].

Although understanding pathophysiology of sepsis is expected to have a marked impact on the development of new effective treatment strategies, underlying molecular mechanisms of renal dysfunction in sepsis are still largely unknown [7]. Furthermore, there are currently no strong biomarkers specifically linked to valid prediction and outcome of sepsis-induced acute kidney failure [27, 28] that could guide a prompt tailored therapy. The exact mechanisms involved are still not clear, due to difficulties to obtain histological data or biochemical kidney function marks in the different sepsis phases [9].

The present study investigates changes of protein expression in kidney tissue in a cecal ligature and puncture (CLP) model of rat sepsis during a time-course of 24 or 48 hours after sepsis induction. More specifically, proteomic analysis was performed between kidney tissues of healthy rats and CLP sepsis rats (24 hours or 48 hours, resp.). These findings were analyzed and compared in the context of bioinformatic protein-interaction analyses. Because proteins are vital molecules in normal biological or pathological functions, analysis of all proteins (i.e., the proteome) may clarify the role of specific proteins and possible protein-protein interactions in AKI [29].

## 2. Materials and Methods

### 2.1. Animal Treatment

Upon approval by the local institutional review board (Regierungspraesidium Karlsruhe, Germany), experiments were performed according to the regulations of the National Institutes of Health Guide for the Use of Laboratory Animals. Male Wistar rats (Charles River Deutschland, Sulzfeld, Germany) were kept under temperature controlled environmental conditions at 22°C on a 14-hour light followed by a 10-hour dark cycle prior to the experiments and were fed a standard diet (Altromin C1000, Altromin, Lage, Germany) with free access to food and water. Animal treatment was performed as previously published in other trials [13–15].

### 2.2. Study Groups

A total of 26 rats weighting 330 ± 10 g standard deviation (SD) were used for this study. Six rats ware assigned to a sham group without cecal ligature and puncture (CLP, control group). Based on mortality results from our laboratory [13], 20 rats were assigned to the sepsis group receiving CLP. Eight rats died before 24 hours and 12 rats were assigned to two different sepsis groups (euthanasia after 24 and 48 hours, resp.) being used for proteomic analysis.

### 2.3. Sepsis Model

For the induction of sepsis, the CLP model [30, 31] was used as previously described [13–15]. On the day of surgery, animals were randomly assigned to the defined sepsis or sham group. Rats were anesthetized under spontaneous breathing with sevoflurane (1 MAC) in a gaseous mixture of 30% oxygen and 70% nitrous oxide.

After weighing, animals of the sepsis group underwent laparotomy and ligation of the cecum (CLP) just below the ileocecal valve with double punctures (18 gauge needle) at the antimesenterial wall. One mm^3^ of feces was manually squeezed out of the intestines in each rat. Thereafter, the abdominal wall was closed in two layers.

Rats in the sham group underwent laparotomy with cecum exenteration but without cecal ligation or puncture. For fluid restoration due to anaesthesia, 10 mL/kg of 0.9% NaCl was injected subcutaneously. In both groups, anaesthesia was terminated, and the rats were allowed to recover. Thereafter, food and water were freely available.

Depending on the group assignment, rats were reanesthetized as described above at the defined time points of the experiments (24 or 48 hours after surgery) and underwent both blood and organ removal after decapitation in deep anaesthesia. After a lateral thoracotomy of both sides the heart was exposed and at least 5 mL of blood was obtained by direct cardiac puncture for bacterial analysis (blood culture BACTECTM PEDS-bottle, cultivation on blood agar, boiled blood agar, and McConkey media, BD PhoenixTM automated microbiology system, Becton Dickinson Diagnostic Systems, Franklin Lakes, NJ, USA), arterial blood gases, electrolytes, haemoglobin (Bayer Vital Diagnostics, Rapidlab 865, Fernwald, Germany), blood cell count (Advia 60, Bayer Vital Diagnostics, Fernwald, Germany), albumin, and phosphate (bromocresol green, ammonium molybdate, Advia 2400, Bayer Vital Diagnostics, Fernwald, Germany). Physiological markers of rats are depicted in Table 1. The kidneys were removed as quickly as possible for proteomic analysis, frozen in isopentane prechilled to −40 to −50°C, and stored at −80°C for further analysis.

### 2.4. Sample Preparation

The following techniques and methodologies to identify altered proteins, including samples preparation, 2D-DIGE, and PMF, were performed by TopLab (Martinsried, Germany), being certified ISO 9001:2008. Briefly, the following steps were performed.

Each frozen kidney was weighed and those from each experimental condition were pooled and grinded with liquid nitrogen in mortar. The tissue powder of the pooled samples was mixed with 4 mL of lysis buffer [7 M urea, 2 M thiourea, 4% CHAPS, 30 mM Tris pH 8.5, Roche complete protease inhibitor cocktail, 1.2% Pefablock SC protease inhibitor, 1% Sigma phosphatase inhibitor cocktail 2, and 1% Sigma phosphatase inhibitor cocktail 3] and transferred into a 15 mL reaction tube. Cells were lysed and proteins dissolved using vigorous vortexing and sonication with a pulse of 1 min on ice. For sonication, portions of ~0.8 mL of each sample were treated with a pulse of 1 min using a Branson Sonifier® Ultrasonic cell disruptor with an ice chilled thermostat. Samples were vortexed, incubated 5 min on ice, and centrifuged for 15 min at 20,000 ×g to pellet debris and insoluble material. The supernatant was taken for further analysis and proteomics.

The protein concentration of samples was determined using a Bradford assay [32]. According to the protein determination results with Bradford assay, 150 *μ*g protein of each sample was diluted with labeling buffer [30 mM Tris, pH 8.5; 7 M urea; 2 M thiourea; 4% CHAPS; Roche complete protease inhibitor cocktail; Pefablock SC protease inhibitor] to a final volume of 450 *μ*L. The samples were rebuffered with labeling buffer using Vivaspin 500 ultrafiltration devices with a cut-off of 5 kDa. After volume reduction samples were present in 100 *μ*L labeling buffer at last with a protein concentration of 5 mg/mL.

### 2.5. DIGE Labeling

65 *μ*g of the samples control and sepsis 48 h treatment was labeled with Cy3 and 65 *μ*g of the samples control and sepsis 24 h treatment was labeled with Cy5. Additionally, 50 *μ*g of each sample was pooled as the internal standard sample (Pool-A) and 65 *μ*g (13 *μ*L) of this mixture was used for each labeling reaction with Cy2. For each labeling reaction with Cy2, Cy3, and Cy5 65 *μ*g protein (13 *μ*L) was applied to 400 pmol CyDye. The reactions were performed on ice for 30 min and stopped by adding lysine and incubating for another 10 min. Labeling was performed according to the manufacturer's protocol (FluoProbes/InterChim:* 2-D DIGE Cy5/3/2 Labeling kit*). The labeling chemistry is based on minimal labeling of lysine residues of the proteins, with one lysine residue labeled per protein on average [33].

After labeling, reactions to run in the same 2D-DIGE gel were pooled. The final volume of each sample was adjusted to 100 *μ*L with lysis buffer B [7 M urea; 2 M thiourea; 4% CHAPS; Roche complete protease inhibitor cocktail; Pefablock SC protease inhibitor] and samples were supplemented with 2% Servalyte and 1% DTT. Finally, 350 *μ*L of rehydration buffer [6 M urea; 2 M thiourea; 2% CHAPS; 1% DTT; 1 v/v% Servalyte 3–10 Iso-Dalt for 2D, Roche complete protease inhibitor cocktail; Pefablock SC protease inhibitor] was added to each sample.

### 2.6. 2D-DIGE

For the 2D-DIGE experiment, samples were loaded directly after labeling onto two 24 cm IPG strips pH 3–10 NL from Serva (*T* = 4%, *C* = 2.7%) using passive in-gel-rehydration for sample application. Therefore, the IPG strips were rehydrated with 450 *μ*L sample for 16 hours at room temperature. Isoelectric focusing (IEF) was performed for 100 kVh in total. All steps were limited with 75 *μ*A per strip and performed at 20°C. The applied instrument was an IEF 100 focusing unit from Hoefer.

Protein separation in the second dimension was carried out on precast 2D HPETM gels (*T* = 12.5%, *C* = 2.7%) with a SDS-GLYCIN-TRIS buffer system overnight (SDS, sodium dodecyl sulfate). A molecular weight standard, commercially available from Serva, was previously labeled with Cy2. The standard comprising masses corresponding to 97, 67, 45, 29, 21, 12.5, and 6.5 kDa. respectively, was applied to the gel and positioned next to the IPG strips. The two 2D-DIGE gels were run in parallel (HPETM FlatTop Tower from Serva with a Multi-TempIII Thermostatic Circulator and a HPE-Power Supply 1500).

### 2.7. Image Analysis and Spot Significance

To visualize the labeled and separated proteins after electrophoresis, the 2D-DIGE gels were scanned at a resolution of 100 *μ*m with a Typhoon FLA 9500 (GE Healthcare).

For image analysis, scan files of the 2D-DIGE gels were loaded into DeCyder 2D software (GE Healthcare, version 7.2). Spots were detected with an estimate of 5,000 spots for the 2D-DIGE gel. Subsequently, a detection area excluding the region of strip application, molecular weight marker, and running front was defined. Spots with a volume below 100,000 were defined to be background. Stained crumbs originated from the Dyes were eliminated by excluding spots with an area below 350. False positive spots, for example, produced by dye artefacts within the gel were removed manually. After the editing the gels were normalized towards the Cy2 channel (internal standard).

### 2.8. Statistical Analysis and Data Handling

Spot IDs were allocated to each spot detected and matched in the two 2D-DIGE gels. An average ratio of spot volumes from different samples was calculated for each spot ID. In case a value of 3.0 was calculated for the average ratio, this means the spot volume did increase three times compared to the reference; an average ratio of −3.0 means a threefold decrease. Unchanged spot volumes have ideally an average ratio of 1.0.

The values of 2-fold SD (twice standard deviation) and the corresponding thresholds of spot volumes were calculated and documented (Table 1). Using filter settings for these threshold values differences in the spot volumes between two samples were identified.

Subsequent image analysis with DeCyder software was performed to determine differences between the samples. The calculation was based on spot volumes after normalization with the signals from the internal standard sample. Based on the total spot number of detected spots and the standard deviation, DeCyder 2D software calculates the threshold of regulation. In case a spot volume of a certain spot in one sample equates the *x*-fold of the spot volume in a second sample, this spot is regulated if *x* is above the threshold of regulation (*x* > +threshold or *x* < −threshold).

Using DeCyder 2D software, the normalized spots were matched and the gel images were grouped according to the samples loaded on the gels. Comparison of the samples was performed using the 2 SD threshold combined with student's *t*-test. Differences between the samples were defined to be significant if the average ratio of the spot volumes was above or below the threshold for 2 SD and the spot did pass the students *t*-test (*P* = 0.01).

### 2.9. Protein Identification

We chose to compare the protein expressions between the samples taken 24 and 48 hours after completion, as well as between sepsis treated samples (grouping the gels of sepsis 24 h treatment and sepsis 48 h treatment) and control sample.

Specific protein spots were identified with peptide mass fingerprinting (PMF) using tryptic in-gel digestion and MALDI-TOF-MS. The spots were manually excised and destained using an acetonitrile containing buffer. In-gel digestion was performed overnight with 0.006–0.02 *μ*g trypsin (Serva, sequencing grade, cat. number 37283) in 10 mM NH_4_HCO_3_ buffer. Peptides were cocrystallized with matrix (10 mg/mL *α*-cyanohydroxy-cinnamic acid) onto the MALDI target. Measurement was performed with a 4800 MALDI-TOF/TOF Analyzer (AB Sciex, Framingham, MA, USA) using positive reflector mode (detection range *m*/*z* 700–4500).

Raw data were processed with GPS Explorer software (AB Sciex, Framingham, MA, USA). All spectra were externally calibrated using a peptide calibration standard. The measured monoisotopic peptide masses were compared to all* Rattus* sequences of the SwissProt database (7,928 rat sequences, updated December 2014) using the software MASCOT (Matrix Science, London, UK).

In order to obtain enough material for analysis with mass spectrometry, a preparative 2D-gel loaded with a mixture of control and sepsis samples of 24 h and 48 h treatment was produced and stained with colloidal Coomassie.

### 2.10. Bioinformatic Network Analysis and Protein Functions Analysis

To identify relevant pathways involved and to describe the interactions and functions of our findings, STRING 10 (Search Tool for Retrieval of Interacting Genes/Proteins, http://string-db.org/), GeneMANIA (http://www.genemania.org/), the KEGG database (Kyoto Encyclopedia of Genes and Genomes, http://www.genome.jp/kegg/), and Pathway Commons (http://www.pathwaycommons.org/) were used.

STRING 10 is a web-server database that provides prediction and information about functional interactions between proteins in form of networks [34, 35]. Predictions are based on systematic genome comparisons that consider conserved genomic neighborhood, gene fusion events, cooccurrence of genes across genomes, and coexpression in other species [36]. Furthermore, published indexed articles, experimental data, and knowledge of manually curated databases are taken into account for the prediction [34].

To assess the likelihood of the prediction (level of confidence), STRING 10 matches and calibrates findings with KEGG pathways database knowledge as a reference [34, 36]. At the end of these events, a score indicates the level of confidence, with the highest confidence set at 0.900, high confidence at 0.700, medium confidence at 0.400, and low confidence at 0.150. This final score results by combination of the scores regarding the other parameters considered (genomic neighborhood, gene fusion, etc.).

Search terms are typed in the box of the web tool, one per line, and the depicted pathway can be adjusted (managing some parameters as level of confidence, number of interactions, etc.) to have the desired view of the interactions and information. Similarly to STRING, GeneMANIA is a tool that helps to predict interactions and function of list of genes in form of network and, when available, of pathway [37, 38]. Algorithms and data sources are different, but the major difference with STRING is that GeneMANIA gives the possibility of better customizing the network, allowing the choice of data sources or highlighting specific functions, with a more comfortable graphic experience [37]. It is developed and continually updated by the University of Toronto and is funded by the Ontario Ministry of Research and Innovation. GeneMANIA knowledge is based on data from large databases, which comprehend Gene Expression Omnibus, BioGRID, EMBL-EBI, Pfam, Ensembl, Mouse Genome Informatics, the National Center for Biotechnology Information, InParanoid, and Pathway Commons [37, 38]. As these software programs use different algorithms, we chose to perform the bioinformatics analysis with all of them in order to retrieve the highest number of predicted interactions, maintaining an acceptable level of confidence (0.400). KEGG, one of the largest existing library of genes and network [39], is organized in three databases: one for the genes of all completed genomes (GENES database); one for functional information, as cellular process, depicted as manually curated functional pathways (PATHWAYS database); and one for enzymes and enzymatic reactions (LIGAND database) [39].

Pathway Commons (PCViz) is a collection of publicly available pathway information from multiple organisms [40]. It provides researchers with convenient access to a comprehensive collection of biological pathways from multiple sources represented in a common language for gene and metabolic pathway analysis. Access is via a web portal for query and download. Pathways can include biochemical reactions, complex assembly, transport and catalysis events, physical interactions involving proteins, DNA, RNA, small molecules and complexes, gene regulation events, and genetic interactions involving genes.

Pathway Commons integrates a number of pathway and molecular interaction databases supporting BioPAX and PSI-MI formats into one large BioPAX model, which can be queried using the web API. This API can be used by computational biologists to download custom subsets of pathway data for analysis or can be used to incorporate powerful biological pathway and network information retrieval and query functionality into websites and software.

### 2.11. Western Blot Analysis

For the 1D SDS PAGE analysis, the specific samples were diluted to a concentration of 10 *μ*g/*μ*L with HPLC water. Subsequently, a further dilution step to 1.5 *μ*g/*μ*L with HPLC water was performed. To each of the six diluted samples an equal volume of 3x SDS sample buffer [30% glycerol; 187.5 mM Tris pH 6.8; 6% SDS, 3% DTT] was added. Samples were boiled for 10 min at 90°C.

For protein separation a precast Tris-Glycine Gel *T*(%) 4–20 from Serva (SERVA*Gel*™ TG 4–20) was used. A total protein amount of 100 *μ*g and 15 *μ*g of each of the three samples was loaded, by applying 20 *μ*L of the prepared dilutions. To estimate the molecular weight of the proteins and to check transfer efficiency 5 *μ*L of a prestained marker (Serva Triple Color protein standard III) and twice 5 *μ*L of an unstained protein marker (Protein test mixture 6, Serva) were also loaded. Lanes without protein load were filled with 1x sample buffer instead to ensure an even gel run.

The gel run was started at low power for 10 min a 10 mA/gel. The main separation took place at 25 mA/gel with the current limited at 500 V. After 110 min (284 Vh) the dye front was about 5 mm above the rear of the gel and the gel run was stopped.

In order to transfer the proteins from the SDS gel onto a polyvinylidene fluoride (PVDF) membrane a Trans-Blot® SD Semi-Dry Electrophoretic Transfer Cell from BioRad and a discontinuous buffer system according to Khyse-Andersen was used. Therefore three sheets of blotting paper (Schleicher & Schuell) and the gel were incubated for 5–10 min in cathodic transfer buffer (0.04 M 6-aminohexanoic acid (epsilon-aminocaproic acid), 20% isopropanol, and 0.01% SDS). One further sheet of blotting paper was incubated in anodic buffer I (0.3 M Tris, 20% isopropanol) and three sheets were incubated in anodic buffer II (0.025 M Tris, 20% isopropanol). The PVDF membrane was activated for 10 s with methanol and then incubated in cathodic transfer buffer.

A glass pipette was carefully used as a rolling pin when assembling the blot to remove air bubbles from the sandwich. For the transfer 5 mA per cm^2^ membrane was applied. The transfer was performed overnight at 4°C. Protein transfer was checked by staining the PVDF membrane with Ponceau S and the blotted gel was additionally stained with colloidal Coomassie to visualize the amount of proteins remaining in the gel after transfer.

For blocking 10% BSA in TBS-T [20 mM Tris pH 7.6; 155 mM NaCl; 0.05% Triton-X 100; 10% BSA] was used and the membrane was incubated for 1 h at RT. Incubation with primary antibody (Anti-COX5B antibody [16H12H9] ab110263) was performed in TBS-T/2% BSA [20 mM Tris pH 7.6; 155 mM NaCl; 0.05% Triton-X 100; 2% BSA] for 42 h at +4°C. The antibody was diluted to 1 *μ*g/mL. Subsequently, the membrane was washed three times for 10 min at RT with TBS-T [20 mM Tris pH 7.6; 155 mM NaCl; 0.05% Triton-X 100]. The secondary antibody (HRP-conjugated Anti-Mouse IgG produced in rabbit, Sigma-Aldrich A9044) was diluted 1 : 100,000 in TBS-T/2% BSA and applied for 1 h at RT. Thereafter the membrane was washed three times for 10 min at RT with TBS-T and once for a few minutes with deionized water. The horseradish-peroxidase-coupled antibody (HRP) was visualized using an enhanced chemiluminescence (ECL) substrate kit from Thermo Fisher (Super Signal West Femto) and the signals were detected with a charge-coupled device (CCD) camera system.

After signal acquisition the membrane was frozen at −20°C until further processing. The next day, the membrane was thawed and stripped from the previously applied antibodies by incubation for 30 seconds with prewarmed stripping buffer [62.5 mM Tris HCl pH 6.8, 2% SDS, and 100 mM DTT]. Subsequently the membrane was extensively washed with water and blocked with 10% BSA in TBS-T [20 mM Tris pH 7.6; 155 mM NaCl; 0.05% Triton-X 100; 10% BSA] for 1 h at room temperature.

As control and to check for equal protein load, anti-alpha tubulin antibody was used as an internal loading control. Incubation with primary antibody (anti-alpha tubulin antibody [EP1332Y], Microtubule Marker, ab52866) was performed in TBS-T/2% BSA [20 mM Tris pH 7.6; 155 mM NaCl; 0.05% Triton-X 100; 2% BSA] for 42 h at +4°C. The antibody was diluted 1 : 50,000. Subsequently, the membrane was washed three times for 10 min at RT with TBS-T [20 mM Tris pH 7.6; 155 mM NaCl; 0.05% Triton-X 100]. The secondary antibody (HRP-conjugated anti-rabbit IgG, Molecular Probes, G21234) was diluted 1 : 7,000 in TBS-T/2% BSA and applied for 1 h at RT. Thereafter the membrane was washed three times for 10 min at RT with TBS-T and once for a few minutes with deionized water. The HRP-coupled antibody was visualized using an ECL substrate kit from Thermo Fisher (Super Signal West Femto) and the signals were detected with a CCD camera system.

## 3. Results

*N* = 26 male Wistar rats were used for the present study. *N* = 6 rats were treated with sham surgery and served as controls (healthy rats), and *N* = 20 underwent CLP for sepsis induction. Of these 20 rats, *N* = 8 died before the experiments and *N* = 6 were sacrificed 24 h after CLP (intermediate sepsis) and *N* = 6 were sacrificed 48 h after CLP (late sepsis). White blood cell (WBC; 3.2*∗*10^9^/*μ*L versus 2.3*∗*10^9^/*μ*L and 2.8*∗*10^9^/*μ*L; *P* < 0.05) count and platelet count (PLT; 818*∗*10^3^/*μ*L versus 400*∗*10^3^/*μ*L and 456*∗*10^3^/*μ*L) differed significantly between the sepsis and control group (Table 1).

### 3.1. 2D-DIGE Analysis

**Figure 1:**
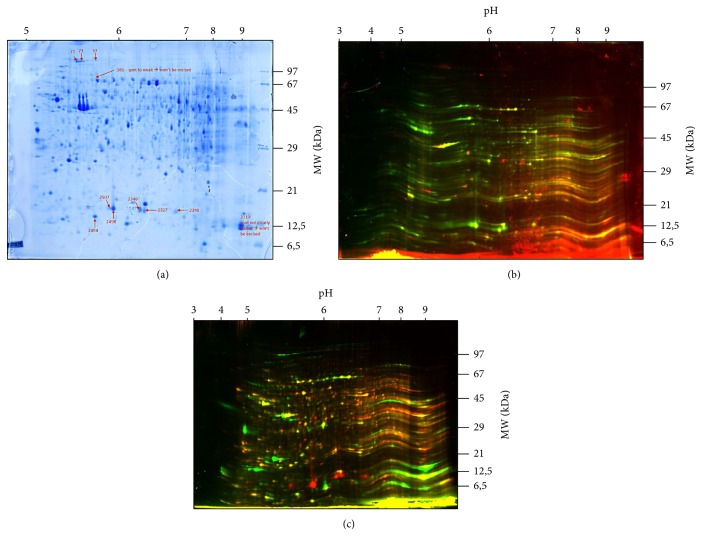
(a) Preparative 2D-Gel (Coomassie blue) of control and sepsis samples. Threshold of regulation is 2 SD (standard deviation) and *t*-test value *P* < 0.01. Spots #381 and #2110 have not been analyzed (Table 2) since they were too weak or not visible for excision. Other spots are marked with arrows and numbers. Alteration of the spots is presented in Table 3. The gel is a sample gel with proteins of the 24 hours' group. (b) Cy3 and Cy5 images of the gels. Cy5: red color = sepsis 24 h group. Cy3: green color = control group. The gel shows proteins of the control group and 24 hours group. (c) Cy3 and Cy5 images of the gels. Cy5: red color = control group. Cy3: green color = 48 h sepsis group. The gel shows proteins of the control group and 48 hours' group.

With the present technique, more than 1.500 different spots were identified on the gels using the 2D-DIGE analysis with DeCyder 2D. After specific calculations by DeCyder 2D, a total of 12 spots was different expressed in the groups/gels analyzed (Figures 1(a)–1(c)).

Seven protein spots were discovered to be differently present between 24 h sepsis and 48 h sepsis. *N* = 5 of these spots were higher and abundant in the 24 h sample sepsis; the other two protein spots were higher and abundant in the 48 h sample sepsis (Table 2). From analysis between sample and the mixture of samples from 24 h sepsis and 48 h sepsis, *N* = 5 different protein spots, all of them in higher abundance in the control sample, were found (Table 2).

### 3.2. Preparative 2D-Gel Analysis

Subsequent analysis with DeCyder 2D of the preparative 2D-gel analysis (grouping the samples of the three groups) found 11 protein spots corresponding to that found with the previous 2-DIGE analyses (Table 2). Of these, two protein spots, ID 381 and ID 2110, belonging to sepsis samples, were not clearly visible and, therefore, were not excised for protein identification. Hence, a total of 9 spots, 4 higher and abundant in control sample, 3 higher and abundant in 48 h sepsis, and 2 higher and abundant in 24 h sepsis, were considered for protein identification by PMF and were matched through MASCOT software with SwissProt database for the identification.

### 3.3. Protein Identification

In the four spots found higher and abundant in the control sample (ID 2346, ID 2507, ID 2327, and ID 2498), major urinary protein (“MUP5” for the STRING database) was identified. MUP5 was detected also in spot ID 2494 that was found higher and abundant in 24 h sepsis. In the other spot overpresent in 24 h sepsis, ID 2494, cytochrome c oxidase subunit B (“COX5B”) was recognized. In three spots found higher and abundant in 48 h sepsis, myosin-6 (“MYH6”) from spots ID 57 and ID 73 and myosin-7 (“MYH7”) from spot ID 71 were identified.  Table 3 shows alterations of protein expression in the specific groups (24 hours or 48 hours) as compared to control. For the five MUP5 spots and COX5B, expression was downregulated at both time points and for MYH6 and MYH7 it was upregulated as compared to control (Figure 3 and Table 3).

### 3.4. Bioinformatic Networks Analyses

STRING 10, GeneMANIA, and PCViz were used for separate network analysis. Using the STRING software, COX5B and MYH6 were found to belong to two separated networks but linked with a level of confidence of 0.508 through cytochrome c oxidase subunit 6 A2 (COX6A2) and actinin alpha 2 (Actn2) (Figure 3, Tables 3 and 4). MUP5 does not present any connection with these networks (Figure 3). COX5B is likely functionally linked to the others subunits of cytochrome c oxidase, NADH dehydrogenase, and ATP synthase, all the connections with a very high level of confidence > 0.990 (Figure 3).

Myosins 6 and 7 were found to be strongly linked to the other proteins of the muscle contraction (troponin I, actin, tropomyosin alpha-1, and alpha-4 chain), all with a level of confidence > 0,960 (Figure 3).

Using GeneMANIA and PCViz for analysis, significant correlation of the identified proteins with other network proteins was found. MYH6 and MYH7 were linked especially to other myosin and contractility proteins (Figures 4 and 5). COX5B was found to be linked to 13 other network proteins (Figure 4) as well as to a human cytochrome c protein (CYCS, Figure 6).

### 3.5. Course of Sepsis: 24 versus 48 Hours after CLP

As compared to the early phase of sepsis (24 hours after CLP), MYH6 (avg. ratio −11.87 and −12.61) and MYH7 (avg. ratio −11.88) were significantly overexpressed in the late phase of sepsis (48 hours after CLP, Table 2). However, MUP (avg. ratio +8.19) and COX5B (avg. ratio +7.67) were less expressed in the late phase of sepsis (Table 2). Localization of COX5B is mainly from the mitochondrion or intercellular space (Figure 7).

### 3.6. Pathways and Signaling Cascades

Using the pathway analysis tool by http://www.genecards.org/, several pathways were identified to be associated with COX5B. Besides respiratory electron transport, oxidative phosphorylation (KEGG data base), and electron transport chain (wiki pathways data base) were most important (Table 4).

### 3.7. Western Blot Analysis and Confirmation of Expression

The protein spot ID2494 (COX5B) was found in the previous 2D DIGE experiment to be significantly lower expressed in the sample sepsis 48 h treatment compared to the samples sepsis 24 h treatment and control. Between the samples sepsis 24 h treatment and control, this protein spot was not significantly differentially expressed.

**Figure 2:**
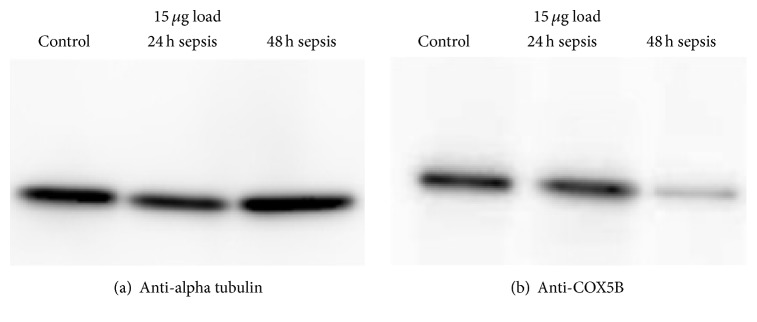
Enhanced chemiluminescence (ECL) signal of Western Blot analysis of anti-alpha tubulin (a) which was used as control and of anti-COX5B (b). The anti-tubulin band is located at 55 kDa and the anti-COX5B band is located at 11 kDa. For analysis, 15 *μ*g load each was used.

**Figure 3:**
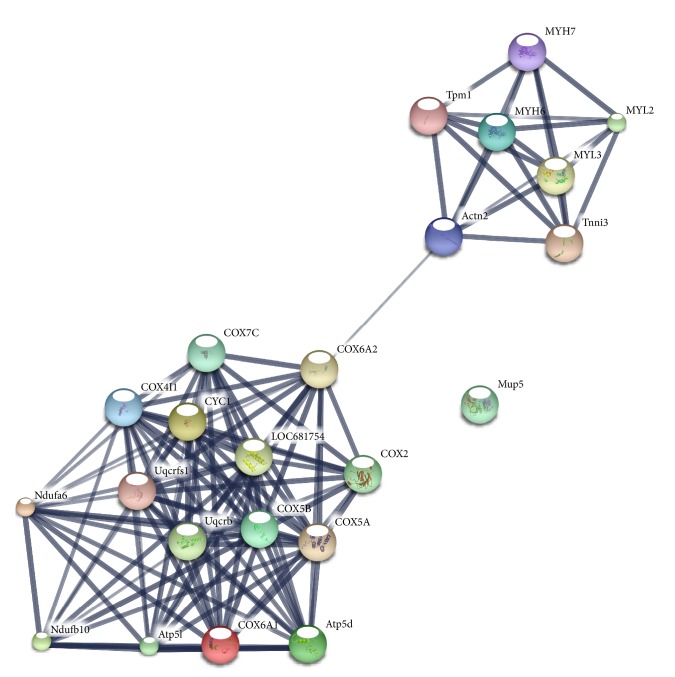
Protein network identified by STRING software (http://www.string-db.org/; confidence view, for MUP5, COX5b, MYH6, and MYH7). Thickness of the lines is directly proportional to the strong of connections among the nodes. Medium level of confidence (score 0.400, with the maximum level of confidence at 0.900 and the lowest at 0.150) and no more than 20 nodes used. For other proteins in the network, see Table 5. To generate the network, all significantly altered proteins were used for analysis, that is, pooled data from the 24 and 48 hours' group.

**Figure 4:**
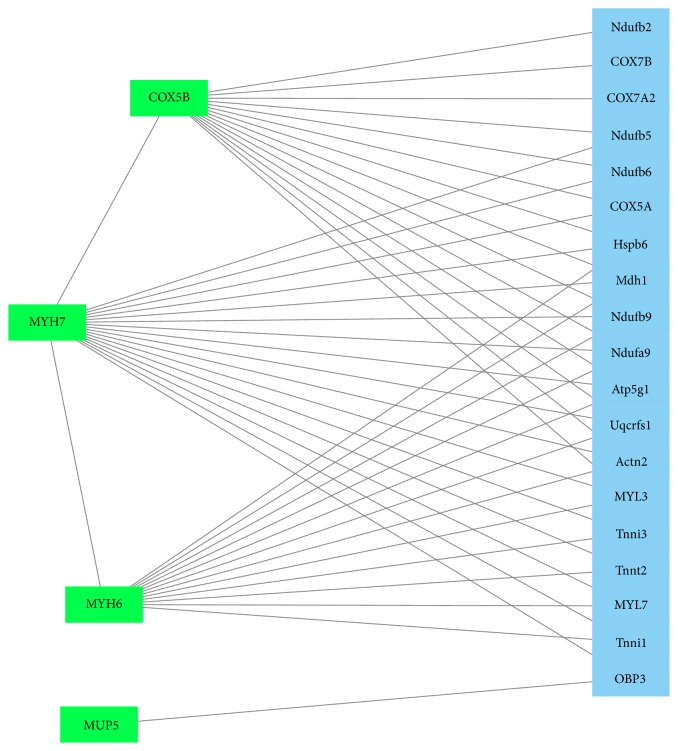
GeneMANIA network (http://www.genemania.org/; visualization using Cytoscape, http://www.cytoscape.org/) found by search of significantly regulated proteins of the present study (i.e., MYH6, MYH7, COX5B, and MUP5). Proteins were entered to gather interactions. Green squares represent searched proteins and blue squares represent connections found by GeneMANIA. The identified proteins MUP5, COX5B, MYH6, and MYH7 are linked together within this network mainly by Atp5g1, COX5a, and OBP3. The lines show interactions between the nodes/proteins. To generate the network, all significantly altered proteins were used for analysis, that is, pooled data from the 24 and 48 hours' group.

**Figure 5:**
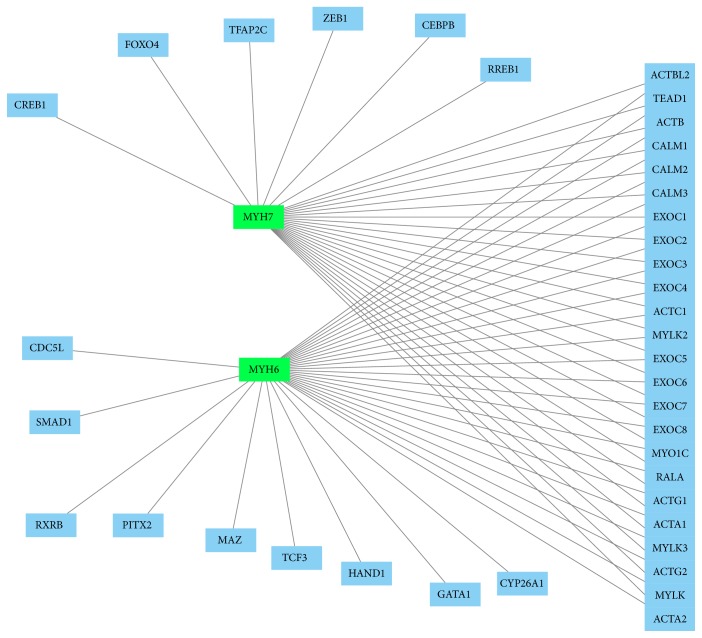
Protein network created by PCViz (Pathway Commons; http://www.pathwaycommons.org/; visualization using Cytoscape, http://www.cytoscape.org/). MYH6 and MYH7 are marked in green. All related proteins (blue nodes) are linked by lines to generate the network. COX5B and MUP5 proteins were not found to be present in this specific network. To generate the network, all significantly altered proteins were used for analysis, that is, pooled data from the 24 and 48 hours' group.

**Figure 6:**
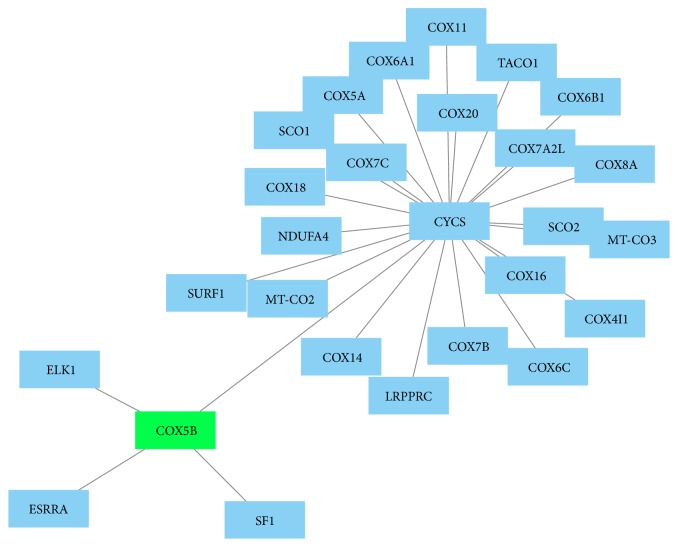
Protein network created by PCViz (Pathway Commons; http://www.pathwaycommons.org/; visualization using Cytoscape, http://www.cytoscape.org/). COX5B, in green, is centered and linked to other proteins via CYCS (human cytochrome c). MYH6 and MYH7 proteins were not found to be present in this specific network. To generate the network, all significantly altered proteins were used for analysis, that is, pooled data from the 24 and 48 hours' group.

Western Blot analysis of the three samples (separated with 1D SDS PAGE and transferred onto PVDF) was performed to check whether the protein COX5B is differentially expressed. After incubation with the anti-COX5B antibody a distinct band at about 12 kDa was detected per lane (Figure 2(b)). No cross-reactions or background signals were observed. Corresponding to the SDS gel analysis, Western Blot analysis also showed a decreased expression of COX5B at 48 hours after sepsis.

As a loading control, the membrane was subsequently incubated with anti-alpha tubulin antibody. A distinct band at about 55 kDa was detected (Figure 2(a)). Following this, the anti-COX5B antibody still gave a clear signal.

The image of the PVDF membrane after transfer and Ponceau S total protein stain showed an even transfer and only slightly different protein load for the three samples as identified by anti-alpha tubulin analysis (Figure 2(a)).

## 4. Discussion

Our findings show differences in expression of several proteins in the rat kidney during intermediate as well as late stage sepsis. Of these proteins, COX5B in particular is of high interest and linked to mitochondrial function and cellular energy production [41]. During the course of sepsis, COX5B was found to be less expressed by both MALDI-TOF and Western Blot analysis in the late phase (i.e., after 48 hours) as compared to the early phase of sepsis.

Proteomics has, during the last decade, increasingly been applied to identify and quantify mitochondrial proteins related to cellular perturbations [12]. So far, several different pathophysiological mechanisms have been proposed for sepsis-induced AKI: vasodilation-induced glomerular hypoperfusion, dysregulated circulation within the peritubular capillary network, inflammatory reactions by systemic cytokine storm or local cytokine production, and tubular dysfunction induced by oxidative stress [2].

### 4.1. CLP Model and Proteomics

There are still two fundamental problems inherently associated with studies of renal responses to sepsis. First, difficult access to the renal tissue and the associated ethical issues make the human research into cellular and molecular biology in critically ill patients with sepsis almost unfeasible [7]. Second, the process of AKI in sepsis involves a complex of multiple dynamically interacting factors and it is clear that both sepsis and septic organ dysfunction are not caused by a single mechanism [7]. Therefore, experimental studies involving animal models are fundamental to better define and characterize the pathophysiological phases of AKI [9].

We chose CLP model for sepsis because it is widely accepted as one of the most close to the pathophysiologic course of sepsis patient [42], it is easily feasible in small animals [30], and it allows a suitable representation of gram-negative sepsis [30]. The decision to analyze samples after 24 and 48 h reflects the evidence that clinical and laboratory signs of sepsis become more conspicuous at 24 h [13, 30], going from a hyperdynamic state (after 12 h) to a progressive hypodynamic phase (after 16–24 h) or late stage (48 h) [30, 43–45].

### 4.2. Course of Sepsis: 24 versus 48 Hours after CLP

Figure 8 shows alterations in expression of the proteins analyzed (Figure 8). The higher abundance of MUP5 in control samples and in intermediate compared to late sepsis samples could suggest a kind of progressive downregulation of this protein with the development of the kidney injury. COX5b also seems to undergo a possible downregulation from intermediate to late sepsis and was confirmed by the Western Blot analysis. MYH6 and MYH7 appear to be the only upregulated proteins during the late stage of sepsis-induced kidney injury.

**Figure 7:**
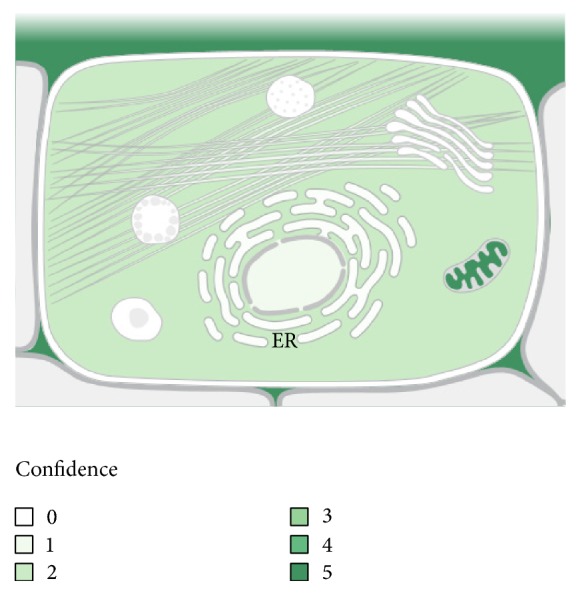
COX5B localizations within a cell (from: subcellular locations, UniProtKB/Swiss-Prot for COX5B Gene). The location of COX5B within the cell is presented with statistic confidence: the darker the green color, the higher the probability for COX5B to be present in this location.

**Figure 8:**
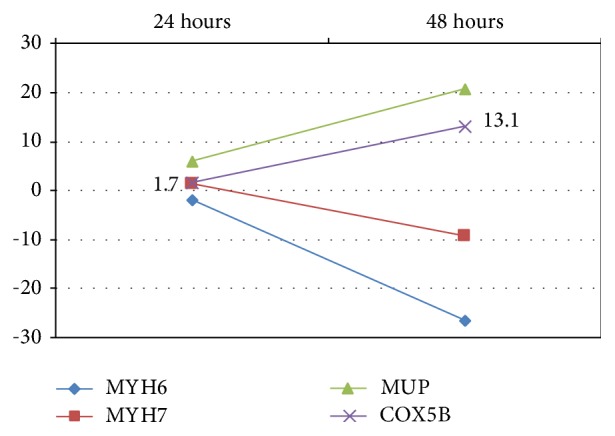
Spot ratios for the proteins identified. A positive value represents lower protein expression during sepsis. A negative value represents higher protein expression during sepsis. Values are shown against control group.

Cytochrome c oxidase (COX, Complex IV) is a mitochondrial electron transport chain enzyme that resides in the mitochondrial inner membrane, and its activity is required to generate the proton motive force that drives downstream ATP synthesis (Figure 7) [46]. It is one of the mitochondrial isoforms of cytochrome c oxidase, that is, the Complex IV of the mitochondrial respiratory chain (Table 6). COX5B is involved in the final step of the oxidative phosphorylation, with the production of H_2_O, and the maintenance of the electrochemical gradient needed to produce ATP. It has been shown that COX5B is implicated in the production of reactive oxygen species (ROS) and nitric oxide (NO) in hypoxic or anoxic conditions in both mammalian and yeast cells [47–50]. ROS can lead to mitochondrial dysfunction, and this condition, with NO overexpression, antioxidant depletion, and reduction of ATP concentration, was found in skeletal muscle cells of sepsis human patients [51] and rats [52].

Association of COX5B with the kidney is not completely new. Tuma et al. also found COX5B being present in the porcine kidney, in both cortex and medulla [6]. Although this study did not analyze proteome alterations during sepsis and in piglets, it showed the importance of COX5B in the kidney. Fedorova et al. found COX4 less expressed in the five-sixth nephrectomy model of chronic renal failure of rats [53]. Expression of COX5B has also been shown to be associated with hypoxia and a low oxygen environment [41].

PGC-1*α* (peroxisome proliferator-activated receptor-*γ*-coactivator-1*α*) is a member of a small family of transcriptional regulators that controls the expression of genes involved in energy homeostasis, mitochondrial biogenesis, fatty acid oxidation, and glucose metabolism [54]. PGC-1*α* is pivotal for the mitochondrial function, as well as for the expression of key mitochondrial proteins [54]. Downregulation of PGC-1*α* has been observed in different experimental models of AKI, and it is implicated as a causative event in renal functional impairment during sepsis-associated AKI [54]. Furthermore, expression of COX5B is regulated by PGC-1*α* [54] and both proteins should be downregulated during emerging kidney injury. It may be speculated that sepsis with consecutive hypotension, altered perfusion, and possible hypoxia could have a comparable effect on COX5B expression.

However, this link between COX5B and PGC-1*α* does not demonstrate validity or superiority of one protein in the context of a biomarker. Future studies should focus on the usefulness of both molecules as biomarkers in AKI.

### 4.3. MYH6 and MYH7

Myosin presence has been demonstrated in kidney descending vasa recta and in renal arterioles and could be involved in the regulation of the blood flow from descending vasa recta to capillary beds [55]. Myosin expressed in nonmuscle tissues plays a central role in cell adhesion, migration, and division [9]. Some other research revealed that MYL12A and MYL12B are crucial for maintenance of the stability of MYH9, MYH10, and MYL6, which leads to normal cell actomyosin function [9].

It has been reported that myosin is implicated in endothelial barrier signaling [56]. Therefore, findings of the present study show that expression of myosin is altered during sepsis and could be a possible explanation for developing acute kidney injury during sepsis. However, it is not clear from the present point of view if the identified myosin originates from the kidney or was retained from filtration by the sepsis-injured kidney but was expressed elsewhere in the body. Furthermore, human skeletal muscle cells exposed to plasma of septic shock patients undergo a muscle protein loss with reduction of myosin, mostly in the early phase of sepsis [57].

### 4.4. MUP5

Also named as “alpha 2-U globulin,” “alpha 2 euglobulin” is a rat protein synthetized in the liver, excreted via the kidneys and reabsorbed by up to 60% by the proximal tubule cells [58]. It is a precursor of a kidney fatty acid-binding protein, involved in mechanism of binding and transport of fatty acids (http://www.uniprot.org/) and it is overexpressed in renal cortex rather than medulla [59]. This protein can strongly bind toxic agents like alkans [58, 60] and is associated with the accumulation of hyaline droplet that leads to damage of the proximal tubular epithelium and renal cancer [61, 62].

In our sample, MUP5 spot was localized at a significantly more alkaline pI than the other analyzed protein spots featuring this protein, suggesting some kind of posttranslational modification and/or degradation of MUP5 between 24 h and 48 h of sepsis.

### 4.5. Mitochondrial Dysfunction during Sepsis

Attempts to understand the regulation of cellular energy production in eukaryotic cells have focused on the components of the mitochondrial respiratory chain and its three sites of energy conservation [63]. Per milligram of tissue, only the heart exceeds the kidney's abundance of mitochondria [64]. The healthy nephron relies on mitochondrially generated ATP in order to facilitate the sodium-coupled reclamation of 99% filtered water [64]. Mitochondrial oxidative phosphorylation is responsible for over 90% of total body oxygen consumption and ATP generation. Segments of the nephron that perform the most chemical work are the proximal tubule and the thick ascending limb of Henle's loop [64].

The respiratory chain (electron-transport chain) includes four individual enzyme complexes (I–IV). These enzyme complexes, notably NADH-ubiquinone oxidoreductase (complex I) and cytochrome C oxidase (complex IV), can be inhibited by reactive oxygen and nitrogen species such as nitric oxide [5].

Cytochrome c oxidase is a key enzyme in the overall regulation of cellular energy production in eukaryotes [63]. Investigation into the underlying cellular mechanisms of AKI increasingly points towards a predominant role of mitochondria [7].

Ultrastructural changes in mitochondria are observed in kidney tubular cells during ischemic nephrotoxic, as well as sepsis-associated AKI, and result in functional decreases [64]. The changes include decreased mitochondrial mass, disruption of cristae, and extensive mitochondrial swelling [64]. These changes in mitochondria are often associated with functional decreases [64]. Mitochondrial dysfunction is, furthermore, also known as an important pathogenic factor in sepsis-associated multiorgan failure, including septic AKI [64]. It is noteworthy that the mitochondria-centered structural and metabolic or bioenergetic alterations occur prior to the onset of AKI, supporting a causative, pathogenic role of mitochondrial damage in this disease condition [64]. Taking into account these aspects, alterations in mitochondrial proteome could serve as potential biomarkers for sepsis-associated AKI.

Mitochondrial injury clearly arises in human sepsis, too, as evidenced by biochemical and structural studies on tissue biopsies obtained in intensive care settings [64]. Despite the recognition of mitochondrial dysfunction in AKI, the underlying mechanism is largely unclear [64]. Using the methodology of the present study, it can give additional insight into affected signaling cascades or tissue pathways. Mitophagy has also been described for the kidney in the course of sepsis [65].

### 4.6. AKI in the Context of the Present Study

In 2012, Hsiao et al. demonstrated a biphasic change of autophagy in the septic AKI (SAKI) model of CLP in rats [66]. Autophagy was shown to elevate at hours but then declined at 9 hours following CLP, where AKI was detected at 18 hours [64].

A considerable number of novel biomarkers for SAKI were developed during recent years and tested in clinical trials, such as cystatin c, neutrophil gelatinase-associated lipocalin (NGAL), interleukin-18 (IL-18), kidney injury molecule-1 (KIM-1), and liver-type fatty acid-binding protein (L-FABP); however, very little of value for earlier prognostication has transpired to date [9]. Therefore, screening of ideal biomarkers with highly sensitivity and specificity for AKI becomes very important and urgent [9]. Besides sepsis, also cis-platin-induced loss of renal function has been shown to be associated with a decreased expression of COX activity [64].

### 4.7. Study Limitations

There are some limitations of the present study which should be clearly addressed. First, validity of CLP model can be critically discussed. We used a standardized approach to induce sepsis and to get stable mortality rates. However, it is not completely clear to what extent rats suffered from acute kidney injury. Second, proteomics as molecular technique has some limitations itself, for example, problematic identification of low-abundant proteins. Analysis was performed standardized with the highest level of quality possible. But, it may be possible that other relevant proteins exist not being identified with the technique used. Third, due to the complex experimental setting and extensive techniques used, the number of rats used seems low. However, standardized approaches were implemented to minimize interrat variations as effective as possible. Fourth, it would be helpful to study the course of COX5B expression in both serum and urine at specific time points. However, since we did not collect urine, we have to refer to future studies to answer this question. Finally, we performed proteomics to identify altered tissue proteins. Therefore, interpretation of these results has to be careful and it has to take into account the fact that unknown mechanisms or protein interactions could exist.

## 5. Conclusions and Future Aspects

The present study analyzed proteome alterations in rat tissue during sepsis to identify possible biomarker candidates in the tissue. In summary, our study combines the power of proteomics with a classic animal model of sepsis.

COX5B could be a promising biomarker candidate since a significant association was found during experimental sepsis in the present study. For future research, COX5B should be evaluated as a biomarker in both human urine and serum to identify sepsis. Furthermore, it might be very interesting to compare this possible biomarker to other already known biomarkers for AKD, for example, PGC1*α* [8]. Unfortunately, it was not the focus of the present study to compare different biomarkers for AKD/AKI but future studies may focus on practicability and usability of different biomarkers in septic patients to find optimal and valid biomarkers.

In the future, it would be highly advantageous to identify biomarkers present in the urine or serum which would reveal AKD very early in its course [29] and to stratify patients and identify patients at risk for AKI. If the renal disease would be detected at a very early stage, reversal or cure might be possible [29].

## Figures and Tables

**Table 1:** Physiological markers of rats in both groups analyzed. Data is presented as means. Fishers' exact test was used for statistical analysis. n.s.: not significant; bold-type numbers are significantly different from control.

Marker	Control/sham group	24-hour sepsis group	48-hour sepsis group	*P* value
CRP, c-reactive protein [ng/*μ*L]	2.0	2.0	2.0	n.s.
WBC, white blood cells [/*μ*L]	3.2	**2.3**	2.8	*P* < 0.05
Ery, erythrocytes	6.1	6.6	6.6	n.s.
Hb, haemoglobin level	13.3	13.2	13.9	n.s.
Hk, hematocrit [%]	0.37	0.38	0.40	n.s.
MCV, mean corpuscular volume	59	58	57	n.s.
MCH, mean corpuscular haemoglobin	22	21	20	n.s.
PLT, platelets [10^3^/*μ*L]	818	**400**	**456**	*P* < 0.05

**Table 2:** Proteins analyzed and identified in the specific groups. Positive value of average ratio: higher expressed in control and sepsis 24 h. Negative value of average ratio: higher expressed in sepsis 48 h. ^*∗*^Probability based Mowse score: Protein scores greater than 52 are significant (*P* < 0.05). Protein scores are derived from ions scores as a nonprobabilistic basis for ranking protein hits. MS: mass spectrometry.

Gel data							MS data					
ID number	2D DIGE			Protein name	UniProt ID, accession number	UniProtKB, gene product ID	Protein score^*∗*^	Sequence coverage%	Peptide count	Total ion score	pI	MW [Da]
	Av. ratio	*t*-test	Comparison									
57	−11.87	0.0043	Sepsis 24 h/sepsis 48 h	Myosin-6 OS = *Rattus norvegicus* GN = Myh6 PE = 1 SV = 2	MYH6_RAT	P02563	360	24%	38	215	5.59	224168
71	−11.88	0.0099	Sepsis 24 h/sepsis 48 h	Myosin-7 OS = *Rattus norvegicus* GN = Myh7 PE = 1 SV = 2	MYH7_RAT	P02564	297	13%	24	246	5.64	223743
73	−12.61	0.0011	Sepsis 24 h/sepsis 48 h	Myosin-6 OS = *Rattus norvegicus* GN = Myh6 PE = 1 SV = 2	MYH6_RAT	P02563	186	12%	25	139	5.59	224168
381	Not identified											
2110	Not identified											
2327	+10.08	0.0065	Control/sepsis	Major urinary protein OS = *Rattus norvegicus* PE = 1 SV = 2	MUP_RAT	P02761	529	78%	16	366	5.85	21009
2340	+8.19	0.0075	Sepsis 24 h/sepsis 48 h	Major urinary protein OS = *Rattus norvegicus* PE = 1 SV = 3	MUP_RAT	P02761	462	76%	15	313	5.85	21009
2346	+7.95	0.0028	Control/sepsis	Major urinary protein OS = *Rattus norvegicus* PE = 1 SV = 4	MUP_RAT	P02761	324	79%	15	178	5.85	21009
2494	+7.67	0.0086	Sepsis 24 h/sepsis 48 h	Cytochrome c oxidase subunit 5B, mitochondrial OS = *Rattus norvegicus* GN = COX5b PE = 1 SV = 2	COX5B_RAT	P12075	81	42%	6	33	7.68	14191
2498	+10.55	00.76	Control/sepsis	Major urinary protein OS = *Rattus norvegicus* PE = 1 SV = 4	MUP_RAT	P02761	364	72%	14	232	5.85	21009
2507	+10.92	00.59	Control/sepsis	Major urinary protein OS = *Rattus norvegicus* PE = 1 SV = 4	MUP_RAT	P02761	244	63%	10	164	5.85	21009

**Table 3:** Identified and significantly regulated protein spots and changes at 24 hours and 48 hours after sepsis induction as compared to control. (↑) = upregulated protein; ↑ = highly upregulated protein; (↓) = downregulated protein; ↓ = highly downregulated protein.

Protein abbreviation	Protein name	Spot number	24 hours versus control	48 hours versus control
MYH6	Myosin-6	57	(↑)	↑
		73	(↑)	↑

MYH7	Myosin-7	71	(↑)	↑

COX5B	Cytochrome c oxidase subunit 5B, mitochondrial	2494	(↑)	↑

MUP5	Major urinary protein	2327	↓	↓
		2340	(↓)	↓
		2346	↓	↓
		2498	↓	↓
		2507	↓	↓

**Table 4:** Proteins, biological processes, molecular functions, and components of the significantly regulated proteins found in the present study (from http://www.ebi.ac.uk/QuickGO/GProtein?ac=P12075, http://www.genecards.org/, and http://www.uniprot.org/uniprot/P02761).

Protein	Biological process	Molecular function	Related pathways	General comment	Component	Alias
COX5B_RAT	Hydrogen ion transmembrane transport Respiratory gaseous exchange	Cytochrome c oxidase activity protein binding metal ion binding	Respiratory electron transport, ATP synthesis by chemiosmotic coupling, and heat production by uncoupling proteins. Metabolism Oxidative phosphorylation Gene expression Alzheimer's disease Respiratory electron transport TP53 regulates metabolic genes	Cytochrome c oxidase (COX) is the terminal enzyme of the mitochondrial respiratory chain. It is a multisubunit enzyme complex that couples the transfer of electrons from cytochrome c to molecular oxygen and contributes to a proton electrochemical gradient across the inner mitochondrial membrane. The complex consists of 13 mitochondrial- and nuclear-encoded subunits. The mitochondrially encoded subunits perform the electron transfer and proton pumping activities. The functions of the nuclear-encoded subunits are unknown but they may play a role in the regulation and assembly of the complex. This gene encodes the nuclear-encoded subunit Vb of the human mitochondrial respiratory chain enzyme. This protein is one of the nuclear-coded polypeptide chains of cytochrome c oxidase, the terminal oxidase in mitochondrial electron transport.	Mitochondrion, mitochondrial envelope, mitochondrial inner membrane, extracellular exosome	Cytochrome c oxidase polypeptide VB, mitochondrial Cytochrome c oxidase Polypeptide Vb Cytochrome c oxidase Subunit Vb COXVB

MUP	Transport/transporter activity	Small molecule binding		Major urinary proteins (Mups) bind and release pheromones. They may also protect pheromones from oxidation. In this context, they play a role in the regulation of social behaviours, such as aggression, mating, pup-suckling, territory establishment, and dominance. Acts as a kairomone, detected by the prey vomeronasal organ and inducing fear reactions in mice.	Extracellular region	

Myh6	ATP metabolic process	ATP binding microfilament motor activity actin binding protein binding	Cell adhesion, integrin-mediated cell adhesion, and migration Cytoskeleton remodeling regulation of actin cytoskeleton by Rho GTPases	Cardiac muscle myosin is a hexamer consisting of two heavy chain subunits, two light chain subunits, and two regulatory subunits. This gene encodes the alpha heavy chain subunit of cardiac myosin. The gene is located ~4 kb downstream of the gene encoding the beta heavy chain subunit of cardiac myosin. MYH6 (Myosin, Heavy Chain 6, Cardiac Muscle, Alpha) is a Protein coding gene. Diseases associated with MYH6 include myh6-related dilated cardiomyopathy and myh6-related familial hypertrophic cardiomyopathy. Among its related pathways are RhoGDI Pathway and PAK Pathway. GO annotations related to this gene include *protein kinase binding* and *ATPase activity*. An important paralog of this gene is MYH4.	Cytoplasm and myosin complex	Myosin, Heavy Chain 6, Cardiac Muscle, Alpha Myosin, Heavy Polypeptide 6, Cardiac Muscle, Alpha (cardiomyopathy, Hypertrophic 1) Myosin Heavy Chain, Cardiac Muscle Alpha Isoform Myosin Heavy Chain 6 MyHC-Alpha

Myh7	ATP metabolic process	ATP binding microfilament motor activity actin binding protein binding		Muscle myosin is a hexameric protein containing 2 heavy chain subunits, 2 alkali light chain subunits, and 2 regulatory light chain subunits. This gene encodes the beta (or slow) heavy chain subunit of cardiac myosin. It is expressed predominantly in normal human ventricle. It is also expressed in skeletal muscle tissues rich in slow-twitch type I muscle fibers. Changes in the relative abundance of this protein and the alpha (or fast) heavy subunit of cardiac myosin correlate with the contractile velocity of cardiac muscle. Its expression is also altered during thyroid hormone depletion and hemodynamic overloading. Mutations in this gene are associated with familial hypertrophic cardiomyopathy, myosin storage myopathy, dilated cardiomyopathy, and Laing early-onset distal myopathy.	Cytoplasm and myosin complex	Myosin, Heavy Chain 7, Cardiac Muscle, Beta Myosin, Heavy Polypeptide 7, Cardiac Muscle, Beta Myosin Heavy Chain Slow Isoform MYHCB

**Table 5:** Proteins found to be involved in the STRING network for the focus proteins Myh6, Myh7, COX5b, and Mup5.

Protein	UniProtKb Id	Extended name and alternative names.	Function	Localization	Score
COX5b	P12075	Cytochrome c oxidase subunit 5b, mitochondrial; cytochrome c oxidase subunit VIA	This protein is one of the nuclear-coded polypeptide chains of cytochrome c oxidase, the terminal oxidase in mitochondrial electron transport	Mitochondrion inner membrane	N/A

Myh6	G3V885	Myosin-6; myosin heavy chain 6; myosin heavy chain, cardiac muscle alpha isoform	Muscle contraction	Cytoplasm; focal adhesion; mitochondrion; muscle myosin complex; myofibril; nucleoplasm; stress fiber; Z disc	N/A

Myh7	G3V8B0	Myosin-7; myosin heavy chain 7; myosin heavy chain slow isoform; myosin heavy chain, cardiac muscle beta isoform	Muscle contraction	Muscle myosin complex; stress fiber; Z disc	N/A

Mup5	P02762	Major urinary proteins; allergen Rat n 1; alpha(2)-euglobin; alpha-2u-globulin; alpha-2u globulin PGCL1	MUPs bind and release pheromones. They may also protect pheromones from oxidation. In this context, they play a role in the regulation of social behaviors, such as aggression, mating, pup-suckling, territory establishment, and dominance. Acts as a kairomone, detected by the prey vomeronasal organ and inducing fear reactions in mice	Cytosol; extracellular space; nucleus	N/A

COX5a	P11240	Cytochrome c oxidase subunit 5A, mitochondrial; cytochrome c oxidase polypeptide Va	This is the heme A-containing chain of cytochrome c oxidase, the terminal oxidase in mitochondrial electron transport	Mitochondrion inner membrane	0.999

COX6a1	P10818	Cytochrome c oxidase subunit 6A1, mitochondrial; cytochrome c oxidase polypeptide VIa-liver	Mitochondrial electron transport	Mitochondrion inner membrane	0.999

Uqcrfs1	P20788	Cytochrome b-c1 complex subunit Rieske, mitochondrial; complex III subunit 5; cytochrome b-c1 complex subunit 5 liver regeneration-related protein LRRGT00195; Rieske iron-sulfur protein; ubiquinol-cytochrome c reductase iron-sulfur subunit	Component of the ubiquinol-cytochrome c reductase complex (complex III or cytochrome b-c1 complex), which is a respiratory chain that generates an electrochemical potential coupled to ATP synthesis	Mitochondrion inner membrane	0.997

Ndufb10	D4A0T0	Protein Ndufb10	Respiratory electron transport	Extracellular exosome; mitochondrial respiratory complex I	0.996

Ndufa6	D4A3V2	NADH dehydrogenase [ubiquinone] 1 alpha subcomplex subunit 6	Accessory subunit of the mitochondrial membrane respiratory chain NADH dehydrogenase (Complex I). Transfer of electrons from NADH to the respiratory chain. The immediate electron acceptor for the enzyme is believed to be ubiquinone	Mitochondrion inner membrane; peripheral membrane protein; matrix side	0.996

LOC681754 (COX6B1)	P80430	Cytochrome c oxidase subunit 6B1; cytochrome c oxidase subunit VIb isoform 1	Connects the two COX monomers into the physiological dimeric form	Mitochondrion intermembrane space	0.995

COX6a2	P10817	Cytochrome c oxidase subunit 6A2, mitochondrial; cytochrome c oxidase polypeptide VIa-heart	This protein is one of the nuclear-coded polypeptide chains of cytochrome c oxidase, the terminal oxidase in mitochondrial electron transport	Mitochondrion inner space	0.995

Atp5d	P35434	ATP synthase subunit delta, mitochondrial; F-ATPase delta subunit	Mitochondrial membrane ATP synthase (F1F0 ATP synthase or Complex V) produces ATP from ADP in the presence of a proton gradient across the membrane which is generated by electron transport complexes of the respiratory chain	Mitochondrion inner space	0.995
Atp5l	Q6PDU7	ATP synthase subunit g, mitochondrial; ATPase subunit g		Mitochondrion inner membrane	0.993

COX4i1	P10888	Cytochrome c oxidase subunit 4 isoform 1, mitochondrial; cytochrome c oxidase polypeptide IV cytochrome c oxidase subunit IV isoform 1	This protein is one of the nuclear-coded polypeptide chains of cytochrome c oxidase, the terminal oxidase in mitochondrial electron transport	Mitochondrion inner membrane	0.991

Uqcrb	Not referenced in UniProtKb	Cytochrome b-c1 complex subunit 7 (Ensembl archive)	Not referenced in UniProtKb		0.991

COX7c	P80432	Cytochrome c oxidase subunit 7C, mitochondrial	This protein is one of the nuclear-coded polypeptide chains of cytochrome c oxidase, the terminal oxidase in mitochondrial electron transport	Mitochondrion inner membrane	0.990

Myl3	P16409	Myosin light chain 3; myosin alkali light chain 1, ventricular; myosin light chain 1, slow-twitch muscle B/ventricular isoform; ventricular myosin light chain 1	Regulatory light chain of myosin, does not bind calcium	A band; I band; myosin complex	0.988

Tnni3	P23693	Troponin I, cardiac muscle; cardiac troponin I	Troponin I is the inhibitory subunit of troponin, the thin filament regulatory complex which confers calcium-sensitivity to striated muscle actomyosin ATPase activity	Contractile fiber; cytoplasm; myofibril; sarcomere; troponin complex	0.987

COX2	P00406	Cytochrome c oxidase subunit 2; cytochrome c oxidase polypeptide II	Cytochrome c oxidase is the component of the respiratory chain that catalyzes the reduction of oxygen to water. Subunit 2 transfers the electrons from cytochrome c via its binuclear copper A center to the bimetallic center of the catalytic subunit 1	Mitochondrion inner membrane; multipass membrane protein	0.985

Cyc1	D3ZFQ8	Cytochrome c-1 (predicted), isoform CRA_c	Electron carrier activity; eme binding	Mitochondrion inner membrane; nucleus	0.985

Tpm1	Q63607	Alpha-tropomyosin 3; tropomyosin 1, alpha, isoform CRA_f; tropomyosin alpha-1 chain	Actin binding; protein homodimerization activity; protein N-terminus binding	Protein complex	0.984

Actn2	D3ZVC0	Protein Actn 2	Calcium ion binding; phosphatidylinositol-4,5-biphosphate binding; thyroid hormone receptor coactivator activity	Extracellular exosome; filopofium; focal adhesion; lasma membrane; Z disc	0.983

ENRSNOG00000050273	MORD80 (obsolete)				0.983

Myl2	D3Z9K3	Myosin regulatory light chain 2, ventricular/cardiac muscle isoform	Cardiac muscle contraction; cardiac myofibril assembly; muscle cell fate specification; muscle fiber development; negative regulation of cell growth; postembryonic development; ventricular cardiac muscle tissue morphogenesis	Actin cytoskeleton; myofibril	0.982

**Table 6:** Pathways associated with COX5B (from: http://www.genecards.org/).

Pathway	Database origin
Electron transport chain	Wiki pathways
Oxidative phosphorylation	Wiki pathways
Respiratory electron transport, ATP synthesis by chemiosmotic coupling, and heat production by uncoupling proteins	Reactome
The citric acid (TCA) cycle and respiratory electron transport	Reactome
Formation of ATP by chemiosmotic coupling	Reactome
Parkinson's disease	KEGG
Huntington's disease	KEGG
Oxidative phosphorylation	KEGG
